# Late-Onset Depression Mimicking a Primary Psychiatric Disorder: Diagnostic Pitfalls in Sporadic Creutzfeldt-Jakob Disease

**DOI:** 10.7759/cureus.105209

**Published:** 2026-03-14

**Authors:** Imane Abourachida, Soukaina Rachidi, Yasmina Zakaria, Mohamed Chraa, Nissrine Louhab

**Affiliations:** 1 Neurology, Mohammed VI University Hospital, Faculty of Medicine and Pharmacy, Cadi Ayyad University, Marrakech, MAR

**Keywords:** elderly patient, late-onset depression, neuropsychiatric symptoms, rapidly progressive dementia, sporadic creutzfeldt-jakob disease

## Abstract

Human prion diseases are rare neurodegenerative disorders that typically present with rapidly progressive neurological decline. Sporadic Creutzfeldt-Jakob disease (sCJD) is a fatal neurodegenerative disorder characterized by rapidly progressive encephalopathy. Although classically presenting with cognitive decline and myoclonus, early manifestations may be predominantly psychiatric, particularly in older adults, leading to diagnostic delay.

We report the case of a 75-year-old woman with no prior psychiatric history who initially developed late-onset depressive symptoms accompanied by behavioral changes and progressive cognitive impairment. Her condition deteriorated over several weeks, evolving into a confusional state with apraxia and agnosia. Initial neurological examination did not reveal myoclonus. Brain magnetic resonance imaging (MRI) demonstrated diffusion-weighted imaging (DWI) hyperintensity involving the basal ganglia with cortical ribboning. Electroencephalography (EEG) revealed generalized periodic sharp wave complexes. Cerebrospinal fluid (CSF) analysis was non-inflammatory, the 14-3-3 protein was positive, and the real-time quaking-induced conversion (RT-QuIC) assay was not performed due to limited local availability. During hospitalization, she progressed to akinetic mutism, generalized myoclonus, autonomic instability, and severe dysphagia, consistent with advanced sCJD.

This case illustrates that isolated late-onset depression may represent an early manifestation of sCJD. In elderly patients presenting with rapidly progressive psychiatric symptoms and poor treatment response, prion disease should be considered. Timely multimodal evaluation, including MRI, EEG, CSF 14-3-3 protein, and RT-QuIC when available, is critical to support the diagnosis and prevent inappropriate psychiatric management.

## Introduction

Sporadic Creutzfeldt-Jakob disease (sCJD) is a rare, fatal prion-related neurodegenerative disorder characterized by a rapidly progressive encephalopathy leading to death within months of symptom onset [1]. Although classic clinical features include rapidly progressive cognitive decline, myoclonus, and cerebellar or pyramidal dysfunction, the presentation can be clinically heterogeneous, contributing to diagnostic challenges [2]. Epidemiological data indicate that sCJD accounts for the majority of human prion diseases, with an annual incidence of approximately one to two cases per million population worldwide [1,3]. The definitive diagnosis requires neuropathological examination, but established diagnostic criteria integrate clinical features with neuroimaging, electroencephalography (EEG), and cerebrospinal fluid (CSF) biomarkers such as 14-3-3 protein and real-time quaking-induced conversion (RT-QuIC) assay results [2,4]. Psychiatric manifestations are increasingly recognized in the early stages of sCJD. Symptoms including depression, anxiety, apathy, and behavioral changes may precede more overt neurological signs, particularly in elderly patients, and can mimic primary psychiatric disorders or late-onset mood disorders. Previous studies have reported that psychiatric symptoms may represent the initial manifestation in approximately 26% of patients and occur within the first 100 days of illness in up to 80% of cases [5,6]. Early identification of red flags such as rapid symptom progression, cognitive decline, and atypical treatment response should prompt neurological evaluation and appropriate investigations. In this context, autoimmune encephalitis represents an important differential diagnosis, particularly because it may initially present with prominent psychiatric symptoms and is potentially treatable when recognized early. We report a case of sCJD revealed by late-onset depressive symptoms, emphasizing the importance of considering neurodegenerative etiologies in elderly patients presenting with new-onset psychiatric manifestations and rapid cognitive deterioration.

## Case presentation

**Figure 1:**
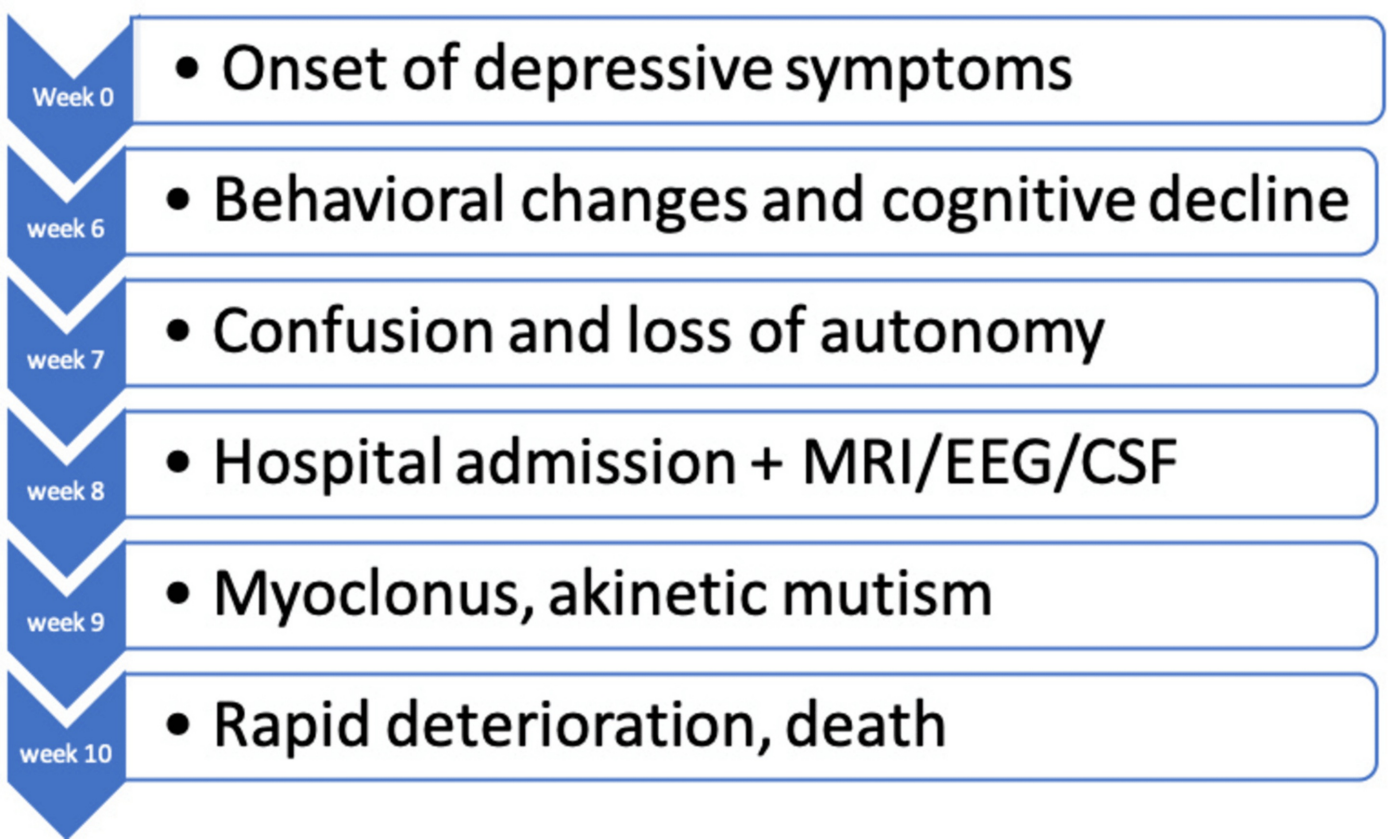
Clinical timeline of symptom progression in sporadic Creutzfeldt-Jakob disease. The figure summarizes the chronological evolution of clinical symptoms and key diagnostic investigations in the present case. Source: The figure was created by the authors using Microsoft Excel (Microsoft Corp., Redmond, WA, USA).

A 75-year-old woman with a medical history of hypertension and suspected paroxysmal atrial fibrillation was admitted to the neurology department of Mohammed VI University Hospital, Marrakech, Morocco, a tertiary referral center, for a rapidly progressive neurocognitive disorder. She had no prior psychiatric history, no history of alcohol or substance use, and no recent exposure to toxins. The patient had no history of neurosurgical procedures, corneal transplantation, dura mater grafting, or treatment with human-derived growth hormone. Approximately two months prior to admission, the patient developed late-onset depressive symptoms characterized by low mood, social withdrawal, and reduced interest in daily activities. She was initially treated with a selective serotonin reuptake inhibitor (SSRI) for approximately four weeks, followed by augmentation with a serotonin-norepinephrine reuptake inhibitor (SNRI), without clinical improvement. Around 20 days before hospitalization, her depressive symptoms worsened significantly and were accompanied by marked apathy, behavioral changes, and disorganized behavior. During this period, she also developed progressive memory impairment and language difficulties. One week prior to admission, her condition rapidly deteriorated, with the onset of confusion and loss of autonomy in activities of daily living. There was no history of fever, seizures, head trauma, or loss of consciousness. On admission, the patient was confused and disoriented in time and space. The chronological progression of symptoms is summarized in Figure 1.

Neurological examination revealed severe cognitive impairment with apraxia, agnosia, and dynamic aphasia. Motor strength was preserved in all four limbs, deep tendon reflexes were symmetrical, and plantar responses were flexor bilaterally. No clinically evident myoclonus was observed at presentation. Vital signs were stable, and there were no signs of meningeal irritation. Cognitive assessment using the Mini-Mental State Examination (MMSE) could not be reliably completed due to poor cooperation, reflecting severe global cognitive dysfunction. Routine laboratory investigations are summarized in Table 1.

**Table 1:** Summary of routine laboratory investigations. Values are presented with corresponding reference ranges. Mild inflammatory marker elevation was noted. ESR: erythrocyte sedimentation rate; CRP: C-reactive protein; ALT: alanine aminotransferase; AST: aspartate aminotransferase; GGT: gamma-glutamyl transferase; TSH: thyroid-stimulating hormone; MCV: mean corpuscular volume

Parameters	Patient values	Reference ranges
Leukocytes (×10^3^/µL)	13.12	4.0-10.0
Erythrocytes (×10^6^/µL)	4.02	4.2-5.2
Hemoglobin (g/dL)	12.6	13.0-16.0
Hematocrit (%)	35.9	37-47
MCV (fL)	89.3	80-100
Platelets (×10^3^/µL)	195	150-400
Neutrophils (%)	95.7	40-75
Lymphocytes (%)	2.6	20-45
ESR (mm/h)	37	0-10
C-reactive protein (mg/L)	7.33	0-5
Sodium (mmol/L)	138.89	135-145
Potassium (mmol/L)	3.62	3.5-5.0
Chloride (mmol/L)	104.64	98-107
Serum creatinine (mg/L)	8.78	7-13
Serum urea (g/L)	0.51	0.15-0.45
Serum glucose (g/L)	1.04	0.70-1.10
ALT (U/L)	17	7-40
AST (U/L)	45	10-40
Alkaline phosphatase (U/L)	36	40-130
GGT (U/L)	12.18	5-40
Albumin (g/L)	38.59	35-50

Routine laboratory investigations revealed inflammatory marker elevation without clinical or microbiological evidence of active infection. These findings were considered non-specific and did not explain the rapidly progressive neuropsychiatric deterioration. Serological testing for human immunodeficiency virus, hepatitis B and C, and syphilis was negative. An extensive autoimmune and paraneoplastic workup, including anti-N-methyl-D-aspartate (NMDA) receptor antibodies and a comprehensive neuronal surface and onconeural antibody panel, was performed and returned negative.

**Figure 2:**
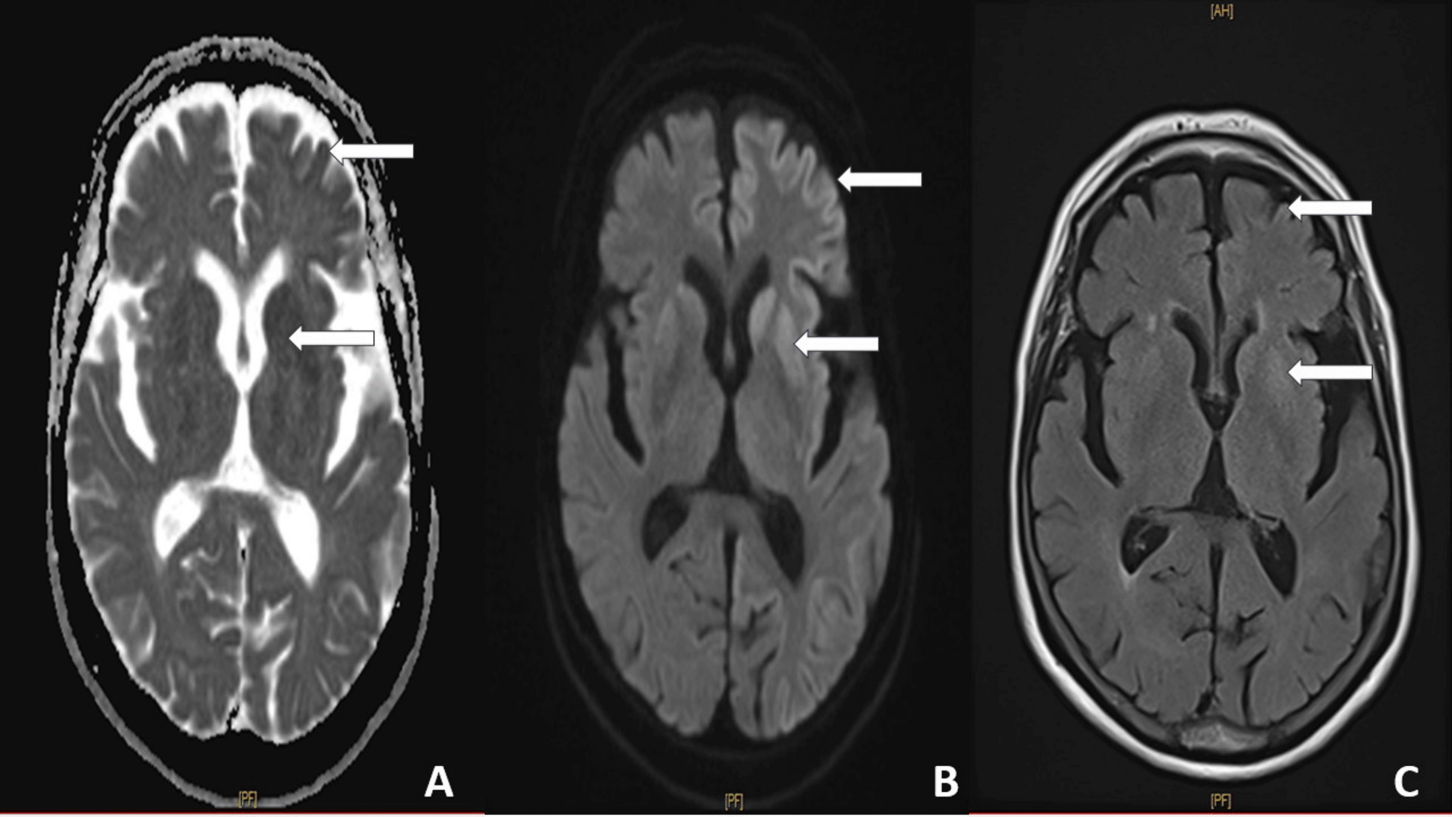
Brain MRI showing characteristic abnormalities in Creutzfeldt-Jakob disease. (A) Apparent diffusion coefficient (ADC) map demonstrating cortical and bilateral striatal hypointensity involving the caudate nuclei and putamina, consistent with true restricted diffusion (arrows). (B) Diffusion-weighted imaging (DWI) showing cortical ribboning and bilateral basal ganglia hyperintensity (arrows). (C) Fluid-attenuated inversion recovery (FLAIR) sequence revealing concordant cortical and striatal hyperintensity without mass effect (arrows). These diffusion abnormalities are considered highly sensitive neuroimaging markers of sporadic Creutzfeldt-Jakob disease and may appear early in the disease course.

Brain MRI showed diffusion-weighted imaging (DWI) hyperintensity involving the basal ganglia and cortical ribboning, as shown in Figure 2.

CSF analysis revealed clear fluid with a normal leukocyte count (3 cells/mm^3^; reference <5) and normal protein concentration (0.25 g/L; reference 0.15-0.45 g/L). Glucose levels were within the normal range (0.75 g/L; reference 0.50-0.80 g/L). Red blood cells were detected at 127 cells/mm^3^, likely reflecting a traumatic lumbar puncture. Microbiological investigations were negative, including Gram stain and bacterial cultures. A multiplex PCR panel for common neurotropic pathogens was also negative. The CSF 14-3-3 protein assay returned positive, supporting the diagnosis in the appropriate clinical context.

**Figure 3:**
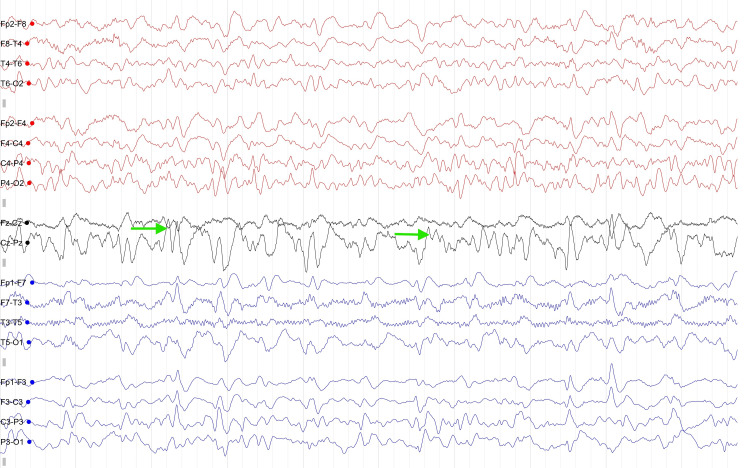
EEG showing periodic sharp wave complexes (PSWC). The EEG demonstrates generalized PSWC occurring at regular intervals on a diffusely slowed background activity. Green arrows indicate representative PSWC, characterized by high-amplitude sharp discharges occurring synchronously across multiple bilateral derivations, a pattern classically associated with sporadic Creutzfeldt-Jakob disease (sCJD). This periodic pattern represents a characteristic electrophysiological finding in sCJD and supports the diagnosis in the appropriate clinical context.

EEG demonstrated generalized periodic sharp wave complexes (PSWC) occurring at regular intervals on a diffusely slowed background (Figure 3).

Given the rapid clinical progression and before the availability of CSF results, empiric intravenous acyclovir was initiated due to concern for possible herpetic encephalitis, despite the absence of fever or focal temporal lobe involvement. The 14-3-3 protein assay subsequently returned positive. The RT-QuIC assay was not performed due to limited local availability. During hospitalization, approximately two months after the onset of initial symptoms, the patient experienced rapid neurological deterioration with the development of generalized myoclonus, akinetic mutism, severe dysphagia, and autonomic instability. Based on the clinical course, characteristic MRI findings, EEG abnormalities, and positive CSF 14-3-3 protein, the diagnosis fulfilled the international criteria for probable sCJD as proposed by Zerr et al. Management was supportive and palliative, and the family was informed of the poor prognosis. The patient died five days after the onset of these advanced neurological manifestations.

This report has inherent limitations related to its single-case design, which restricts generalizability. Additionally, neuropathological confirmation was not available, and the RT-QuIC assay could not be performed. Although the diagnosis fulfilled international criteria for probable sCJD, the absence of definitive pathological confirmation represents a diagnostic limitation.

## Discussion

Early manifestations of sCJD may be clinically misleading, particularly when psychiatric symptoms dominate the initial presentation. Several studies have demonstrated that diagnostic sensitivity varies across the clinical spectrum of sCJD, with reduced diagnostic yield during early stages when classical neurological signs are subtle or absent [2]. This heterogeneity contributes significantly to diagnostic delay, especially in patients initially presenting with affective or behavioral symptoms.

Psychiatric manifestations are increasingly recognized as part of the early clinical spectrum of sCJD [2]. Depression, anxiety, apathy, irritability, and behavioral disturbances may precede overt neurological deterioration by weeks or months, particularly in elderly patients [7,8]. Retrospective analyses indicate that isolated psychiatric symptoms may initially lead to misdiagnosis as primary psychiatric illness, delaying neurological referral [2]. In our patient, late-onset depression occurred in the absence of prior psychiatric history and was marked by treatment resistance, both of which should be considered early warning signs.

The diagnostic challenge is compounded by the overlap between psychiatric-onset sCJD and other causes of rapidly progressive dementia (RPD). Reviews of RPD emphasize that early sCJD may resemble primary psychiatric disorders or other neurodegenerative conditions before classical neurological features emerge [7]. In this context, the rapid progression of symptoms over weeks rather than years represents a key clinical clue. Although the initial depressive phase in our patient evolved over approximately two months, a marked neurological acceleration occurred during the final weeks, consistent with the classical rapidly progressive trajectory of sCJD [3].

Neuroimaging plays a pivotal role in overcoming these diagnostic pitfalls. Brain MRI, particularly DWI, demonstrates high sensitivity and specificity for sCJD, frequently revealing cortical ribboning and basal ganglia hyperintensities even early in the disease course. Reported DWI sensitivities exceed 90% in several series, often outperforming EEG and conventional CSF biomarkers in early-stage disease [8].

EEG remains an important adjunctive investigation. Periodic sharp-wave complexes, while not universally present, demonstrate high specificity for sCJD and may support the diagnosis when identified [9]. However, EEG abnormalities may appear later in the disease course, limiting their sensitivity in early presentations.

CSF biomarkers further contribute to diagnostic accuracy. The detection of 14-3-3 protein reflects rapid neuronal injury and supports the diagnosis when interpreted alongside compatible clinical and imaging findings. Reported sensitivities of CSF 14-3-3 range from approximately 85% to 92%, although specificity is lower, as elevated levels may also occur in other rapidly progressive neurological conditions such as stroke, encephalitis, or paraneoplastic syndromes [10].

More recently, RT-QuIC has emerged as a highly specific diagnostic assay for prion diseases. RT-QuIC directly detects misfolded prion protein and demonstrates specificities exceeding 98% and sensitivities generally above 90% in sporadic CJD, significantly reducing false-positive results compared with 14-3-3 testing [10]. Its incorporation into updated diagnostic criteria has substantially improved diagnostic confidence, particularly in atypical or psychiatric-onset presentations [11].

According to the internationally accepted diagnostic criteria for sCJD, our patient fulfilled the definition of probable sCJD. She presented with a rapidly progressive cognitive decline followed by the development of myoclonus and akinetic mutism, two core clinical features included in the diagnostic criteria. In addition, supportive investigations revealed characteristic diffusion-weighted MRI abnormalities involving the cortex and basal ganglia, PSWC on EEG, and positive CSF 14-3-3 protein, all of which strongly support the diagnosis. In the present case, RT-QuIC testing was not available due to limited local resources, and the diagnosis relied on characteristic MRI findings, compatible clinical progression, EEG abnormalities, and supportive CSF analysis. This underscores the ongoing diagnostic challenges faced in resource-limited settings. 

Despite improvements in diagnostic strategies, the natural history of sCJD remains characterized by rapid neurological deterioration leading to akinetic mutism and death within months of symptom onset [5].

Although no disease-modifying therapy is currently available, recent experimental approaches such as passive immunotherapy targeting the prion protein have demonstrated biological feasibility, although without proven clinical benefit in advanced disease stages to date [11].

Finally, ongoing national and international surveillance networks remain essential for early recognition of atypical presentations, monitoring epidemiological trends, and mitigating potential public health risks associated with prion diseases [12].

Overall, this case highlights that early sCJD may initially present with psychiatric symptoms, creating significant diagnostic pitfalls. Careful attention to rapid progression, early cognitive decline, and prompt use of diffusion-weighted MRI and appropriate CSF assays is essential to reduce diagnostic delay and avoid misattribution to primary psychiatric disorders.

## Conclusions

This case highlights the diagnostic challenges of sCJD, particularly in elderly patients presenting with atypical and predominantly psychiatric symptoms. In our patient, late-onset depression preceded overt neurological signs, contributing to an initial misattribution to a primary psychiatric disorder. Subsequent rapid clinical deterioration prompted further investigations, and infectious, autoimmune, and paraneoplastic etiologies were excluded. Diffusion-weighted MRI represented the earliest and most informative diagnostic investigation, revealing characteristic cortical ribboning and basal ganglia abnormalities. When interpreted in the context of rapidly progressive cognitive decline, these findings prompted further evaluation with EEG demonstrating PSWC and CSF analysis showing positive 14-3-3 protein, collectively supporting the diagnosis of probable sCJD. Clinicians should maintain a high index of suspicion for prion disease in elderly patients presenting with rapidly progressive psychiatric symptoms, particularly when accompanied by early cognitive decline and poor response to treatment, and diffusion-weighted MRI should be considered an early key investigation in this diagnostic pathway.
